# ﻿*Eriobotryacrassifolia* (Rosaceae), a new species from Yunnan Province, China

**DOI:** 10.3897/phytokeys.214.96425

**Published:** 2022-11-22

**Authors:** Kai-Kai Meng, Su-Fang Chen, Min Lin, Wen-Bo Liao, Jian-Hua Jin, Qiang Fan

**Affiliations:** 1 State Key Laboratory of Biocontrol, School of Life Sciences, Sun Yat-sen University, Guangzhou 510275, China Sun Yat-sen University Guangzhou China; 2 Guangdong Provincial Key Laboratory of Plant Resources, School of Life Sciences, Sun Yat-sen University, Guangzhou 510275, China Sun Yat-sen University Guangzhou China

**Keywords:** *Eriobotrya*, new species, molecular evidence, morphological traits

## Abstract

The new species *Eriobotryacrassifolia*, collected from Yunnan Province, China, is characterised and illustrated. A phylogeny based on chloroplast genomes supported its closest affinity with *E.tengyuehensis*, while a phylogeny based on 197 single-copy nuclear genes supported its closest affinity with *E.fragrans* and *E.deflexa*. Morphologically, however, it resembles *E.angustissima*. Nevertheless, it can be easily distinguished from *E.angustissima* by its abaxially retroflexed and sharply serrate leaf margins, densely rusty tomentose inflorescences, and oblong or elliptic leaves.

## ﻿Introduction

The genus *Eriobotrya* Lindley (Rosaceae, tribe Maleae) is an economically important genus, widely distributed across the tropical and subtropical regions of East Asia (Chen et al. 2022). It consists of around 30 species, with 14 species distributed in China (Yü et al. 1974; Gu and Spongberg 2003). During the past decade, a total of four new *Eriobotrya* species have been reported and described, including *E.fulvicoma* W.Y. Chun *ex* W.B. Liao, F.F. Li & D.F. Cui (Li et al. 2012), *E.condaoensis* X.F. Gao, M. Idrees & T.V. Do (Idrees et al. 2018), *E.laoshanica* W.B. Liao, Q. Fan & S.F. Chen (Chen et al. 2020) and *E.shanense* D.H. Kang, H.G. Ong & Y.D. Kim (Kang et al. 2021).

Recently, intergeneric delimitation between *Eriobotrya* and *Rhaphiolepis* has been greatly disputed, based on the questions of whether they are both monophyletic or whether the latter is nested within the former. Liu et al. (2020a) argued that *Eriobotrya* and *Rhaphiolepis* should be merged into one genus, based on the evidence from chloroplast genomes, as well as the entire nrITS, which strongly supported the paraphyly of *Eriobotrya*, with *Rhaphiolepis* nested within it. However, based on the partial ITS region, the phylogenetic trees inferred from Bayesian Inference (BI) and Maximum Likelihood (ML) demonstrated that *Eriobotrya* is monophyletic with robust support and could be distinct from *Rhaphiolepis* (Kang et al. 2021). In the latest research (Dong et al. 2022), with increased sampling and well-supported phylogenies, based on nrITS datasets, the monophyly of *Eriobotrya*, as well as *Rhaphiolepis*, was also supported. Based on our recent work (Chen et al. 2022), the plastome data, indeed, agree with the paraphyly of *Eriobotrya*, while simplified haplotype networks of three nuclear genes of *PXMP2*, *TPP2* and *C23H* indicated that all haplotypes of *Rhaphiolepis* formed one distinct clade and the phylogenetic tree constructed from 197 nuclear genes revealed that *R.philippinensis*, as a representative of *Rhaphiolepis*, was placed outside all *Eriobotrya* species with high support. Besides, morphologically, the (circular) annular ring after sepal senescence could only be observed in *Rhaphiolepis* regardless of the difference in persistent sepals of *Eriobotrya* (Robertson et al. 1991; Gu and Spongberg 2003). Interspecific and intergeneric chloroplast capture might be frequent in Maleae (Liu et al. 2020b; Chen et al. 2022). Therefore, we suggest that ancient chloroplast capture once occurred in the ancestor of *Rhaphiolepis*, which captured the chloroplast of *Eriobotrya* species, thus causing the cyto-nuclear discordance. Here, we propose that both *Eriobotrya* and *Rhaphiolepis* are monophyletic and these two genera should not be merged.

Previously, we described a new species of *Eriobotryalaoshanica* that flowers in autumn in the Laoshan Natural Reserve, Malipo County, south-eastern Yunnan Province, China (Chen et al. 2020). Afterwards, through our numerous field investigations, we identified another new sympatric *Eriobotrya* species that flowers in spring. After careful comparison and verification, we confirmed it as a new species, which is described and illustrated here.

## ﻿Materials and methods

Field investigations and observations were conducted during the flowering and fruiting periods of the putative new species. Fresh leaves were collected and stored in silica gel for molecular experiments. Herbarium specimens were used for further morphological comparisons. Voucher specimens were deposited in the herbarium of Sun Yat-sen University (**SYS**). Total DNA extraction, sequencing and data analyses were conducted following procedures described by Chen et al. (2022).

We retrieved all available chloroplast genomes of *Eriobotrya* and *Raphiolepis* from the NCBI public database (https://www.ncbi.nlm.nih.gov/). In total, we downloaded 58 accessions representing 24 *Eriobotrya* species, eight *Raphiolepis* species and five related species as outgroups. Chloroplast genome sequences were aligned using MAFFT v.7 (Katoh and Standley 2013). The alignment was then trimmed using trimAl v.1.2 by setting the “gappyout” model (Capella-Gutiérrez et al. 2009). The Maximum Likelihood (ML) tree was constructed using IQ-TREE v.1.6.8 under 5,000 replicates of the SH approximate likelihood ratio test (SH-aLRT) and 10,000 replicates of ultrafast bootstraps (UFBS) (Nguyen et al. 2015). For the phylogenetic tree based on nuclear genes, we referred to the results of Chen et al. (2022), in which 197 single copy nuclear genes were used and one accession of the new species, 35 accessions of other *Eriobotrya* species, one accession of *Rhaphiolepis* and five accessions of outgroups were included.

## ﻿Results

The alignment length of the chloroplast genome was 156,859 bp, amongst which 1,736 sites were parsimony-informative. According to the Bayesian Information Criterion (BIC), the best-fit nucleotide substitution model was GTR+F+I+G4.

**Figure 1.**
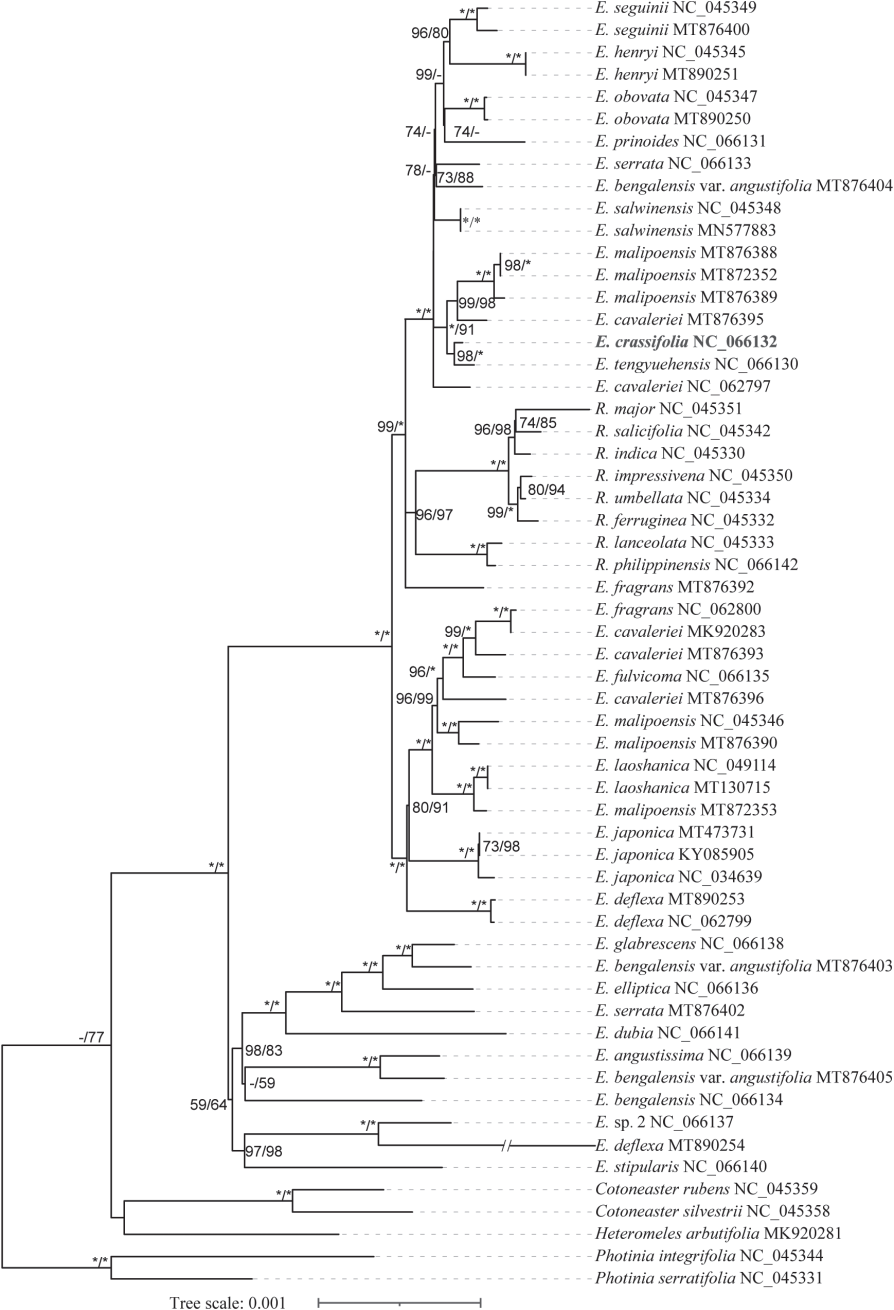
The phylogenetic tree, based on 58 complete chloroplast genomes. Numbers near nodes indicated SH approximate likelihood ratio test (SH-aLRT) and ultrafast bootstrap support values (UFBS), respectively. The symbols “*” imply that SH-aLRT ≥ 95% or UFBS ≥ 95%. The symbols “-” imply that SH-aLRT < 50% or UFBS < 50%. The new species was highlighted in bold.

The phylogenetic tree inferred from chloroplast genomes demonstrated that this new species is sister to *E.tengyuehensis* with high support (SH-aLRT = 98; UFBS = 100, Fig. 1), but distantly related to *E.fragrans* and *E.deflexa*. However, based on the 197 single copy nuclear genes, the species tree showed that this new species was closely related to *E.fragrans* and *E.deflexa* with low support (quartet score, QS = 38; local posterior probabilities, Astral-PP = 0.74) (fig. 5b in Chen et al. 2022), while the divergence time estimation indicated that it was sister to the sympatric species of *E.laoshanica* (fig. 8b in Chen et al. 2022), in which this new species was marked with *E.* sp1. All this molecular evidence identified that *E.angustissima* was distantly related with the new species although they were similar in morphology.

## ﻿Discussion

Based on the PhyloNet analysis of Chen et al. (2022), we considered that *E.tengyuehensis* might originate from the hybridisation of *E.obovata* and this new species, with the latter as its female parent. It is for this reason that *E.tengyuehensis* and the new species formed a well-supported sister clade in the chloroplast phylogeny. Based on the species tree, this new species was closely related with *E.fragrans* and *E.deflexa*, but the support values were very low. As concluded by Chen et al. (2022), extensive ancient hybridisation events occurred in this genus during species diversification. Hence, this new species might also be involved in ancient hybridisation events and its parents were not found in extant species. Morphological traits of the parents were also unknown and we supposed morphological similarity between this new species and *E.angustissima* was just a coincidence. Besides, population genomics of *Eriobotrya* species, especially for those distributed in Yunnan Province, need to be further conducted to explore the potential causes of those discordances.

Geographically, *E.fragrans* is distributed in Guangdong and Guangxi Provinces, China and *E.deflexa* is distributed in Guangdong and Taiwan Provinces, China. Additionally, they are also significantly different from this new species in morphology. Though this new species shares the same distribution regions with *E.laoshanica*, they are distinctly different, both in phenology and morphology. The new species is morphologically similar to *E.angustissima* and *E.tengyuehensis*. However, by our comprehensive comparisons, it could be easily distinguished from *E.angustissima* by its oblong or elliptic (vs. narrowly oblong) and thickly (vs. thinly) coriaceous leaves; inflorescence densely rusty tomentose (vs. glabrous or glabrescent) and 10–17 lateral veins (vs. 8–10). Though this new species shares a few characteristics with *E.tengyuehensis*, for example, thickly coriaceous leaves, similar number of lateral veins and styles, it could be easily distinguished from the latter by its abaxially retroflexed and sharply serrate margin (vs. not retroflexed and entire basally, serrate apically) and much smaller leaves (9–11 × 2.5–3.5 cm vs. 12–17 × 5–7 cm) (Table 1).

**Table 1.** Morphological comparisons amongst *Eriobotryacrassifolia*, *E.tengyuehensis* and *E.angustissima*.

Characters	*E.crassifolia*	*E.angustissima*	*E.tengyuehensis*
Leaf shape and size	oblong or elliptic, 9–11 × 2.5–3.5 cm	narrowly oblong or very narrow linear-lanceolate, 5–10 × 1–2 cm	oblong, elliptic or nearly obovate, 12–17 × 5–7 cm
Leave margin	abaxially retroflexed, sharply serrate	abaxially slightly retroflexed, entire basally, serrate apically	abaxially not retroflexed, entire basally, serrate apically
Texture of leaves	thickly coriaceous	thinly coriaceous	thickly coriaceous
Lateral veins	10–17 pairs	8–10 pairs	10–15 pairs
Inflorescences	densely rusty tomentose	glabrous or glabrescent	brownish-yellow tomentose
Petal shape	obovate-triangular or suborbicular	obovate or obcordate	obovate
Fruit shape and size (diameter)	oblong or subglobose, 6–7 mm	subglobose, 10 mm	subglobose, 7 mm
Styles	2–4	3–5	2–3

### ﻿Taxonomic treatment

#### ﻿ Eriobotrya crassifolia

Taxon classificationPlantaeRosalesRosaceae

Q.Fan, S.F.Chen & K.K.Meng sp. nov.

E7A3753C-2668-50DF-84D5-FCB9CD44C42F

urn:lsid:ipni.org:names:77308552-1

##### Type.

**Figure 2.**
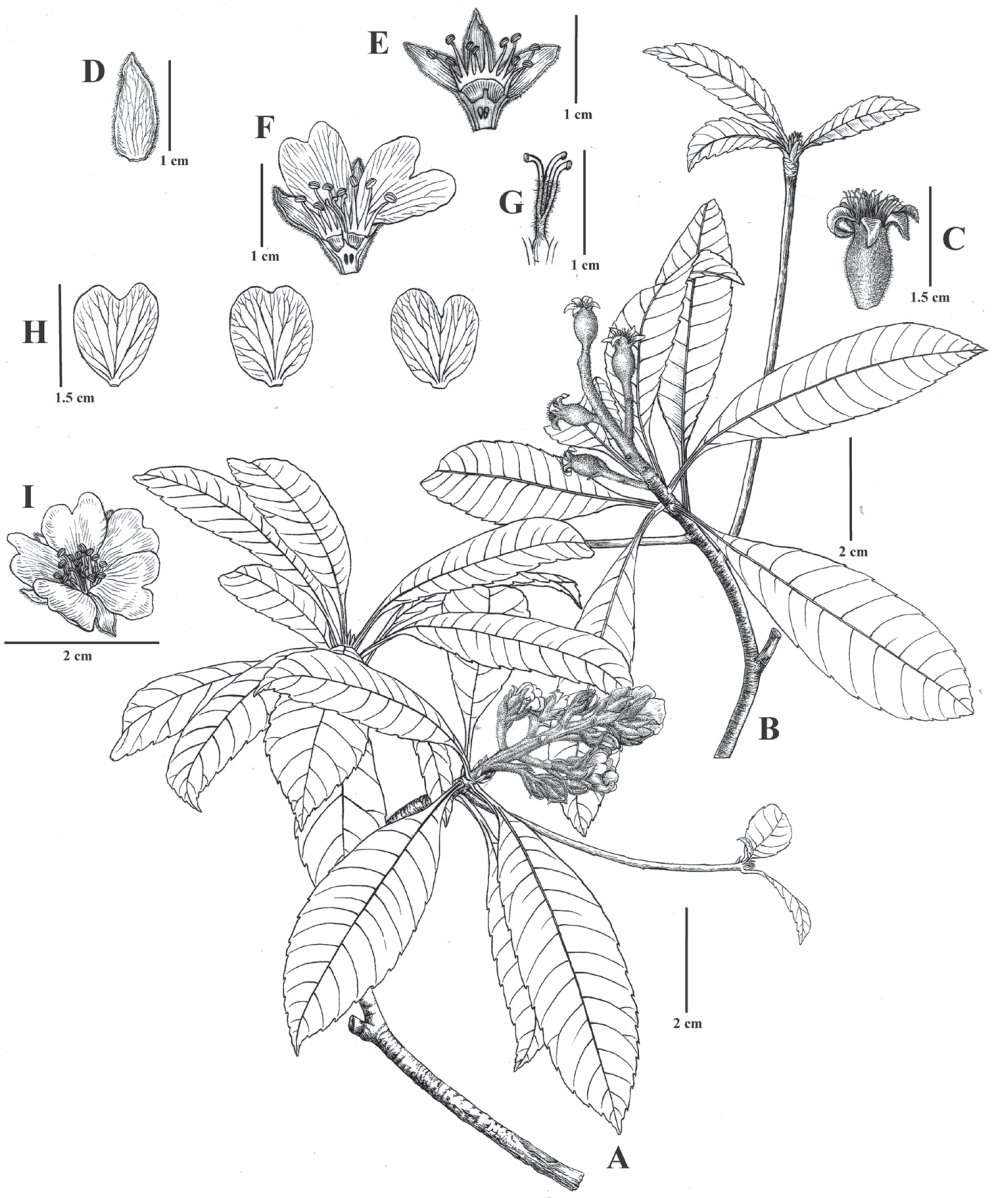
*Eriobotryacrassifolia* sp. nov. **A** flowering branch **B** fruiting branch **C** young fruits with persistent calyx lobes **D** calyx lobe **E** flower in longitudinal section, showing the ovary, stamen and calyx lobes **F** flower in longitudinal section, showing the pistil, stamens and petals **G** styles **H** petals **I** flower in front view. Illustrated by Yun-Xiao Liu.

**Figure 3.**
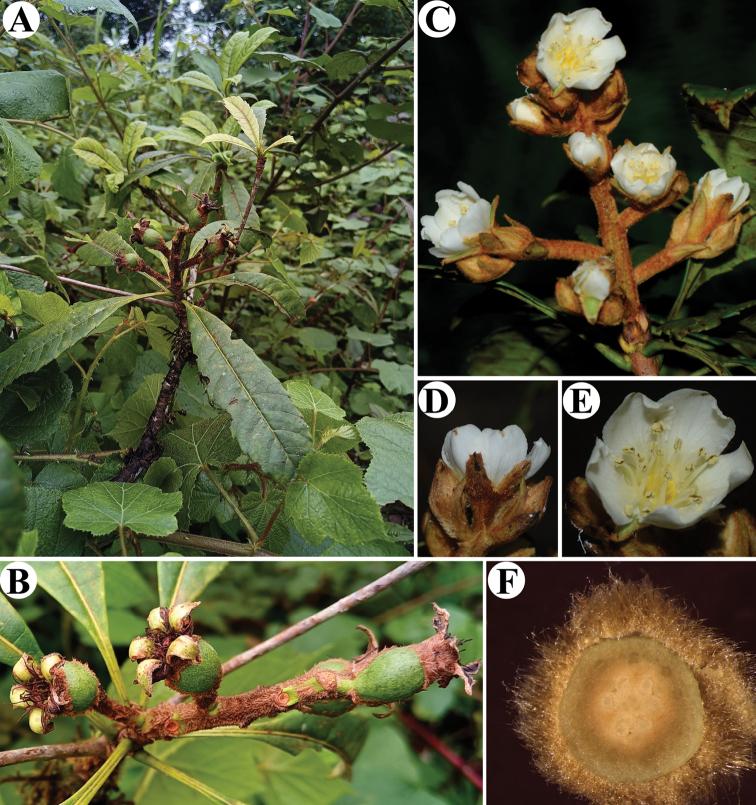
*Eriobotryacrassifolia* sp. nov. **A, B** fruiting branch **C** flowering branch **D** flower in side view **E** flower in front view **F** ovary in transverse section, showing the 3-loculed ovary. Photographed by Qiang Fan.

China. Yunnan Province, Malipo County, Xiajinchang Town, Mount Laoshan, in thick forests on the slopes of limestone hills, 23°07'N, 104°50'E, 1684 m a.s.l., 21 March 2022, *Q. Fan* 19220 (holotype: SYS; isotype: IBSC, SYS) (Figs 2, 3).

##### Diagnosis.

The new species resembles *E.angustissima*, but differs from the latter by its leaf shape and texture, the number of lateral veins and the indumentum of inflorescence.

##### Description.

Evergreen trees, 15–25 m tall; stems 10–30 cm in diameter; branchlets grey-white, terete, glabrous, 4–8 mm in diameter. Leaves spirally inserted on branches and often crowded at tips of branchlets, margin sparsely serrate; petioles 1–1.5 cm long, glabrous; stipules linear-lanceolate, 10–12 × 1–2 mm, glabrous; leaf blades oblong or elliptic, 9–11 × 2.5–3.5 cm, thickly coriaceous, glabrous, mid-vein prominent, raised abaxially, lateral veins 10–17 pairs, sporadically dichotomous before terminating at margin, apex acute to acuminate, base cuneate, margin deflexed with sharply serrate. Inflorescence in terminal panicles, 17- to 30-flowered, 6.7–9.8 × 4–7.7 cm, with 4–7 lateral racemes, peduncle and pedicels densely rusty tomentose, pedicels 3–4 mm; bracts and bracteoles ovate-triangular, 2–4 × 8–10 mm, abaxially densely tomentose, adaxially glabrous; petals white, quincuncial, obovate-triangular or suborbicular, 9–10 × 6–8 mm, apex emarginate; stamens 20; filaments 5.3–8.1 mm long, glabrous; anthers 1.4–1.5 mm long; styles 6.2–9 mm long; ovary inferior; hypanthium shallow-cupular, 9–10 × 6–8 mm, abaxially densely rusty tomentose, 5-lobed, the calyx lobes triangular-ovate, 9–10 × 5–6 mm, abaxially densely rusty tomentose; ovary 2–4-loculed, with 2 ovules per locule; styles 2–4, mostly 3, densely yellowish villous in the lower part, connate at base or fused at one fourth of the base; fruits elliptoid or subglobose, 6–7 × 7–9 mm, glabrescent, capsules crowned by five persistent calyx lobes; seeds 2–3 per fruit.

##### Phenology.

Flowering from March to April, fruiting from June to August.

##### Etymology.

Latin *crassus*, thick, and *folia*, leaved, alluding to leaf thickness

##### Distribution and habitat.

Presently, *Eriobotryacrassifolia* is known from a single locality, Laoshan Natural Reserve, Malipo County, south-eastern Yunnan Province, China. It is distributed in thick forests on the slopes of limestone hills at altitudes of 1502–1684 m a.s.l.

##### Conservation status.

Only two populations were found with no more than 200 mature individuals. Thus, the species status could be considered as Endangered (EN; D), according to the IUCN Red List Criteria (IUCN Standards and Petitions Subcommittee 2022).

##### Additional specimens examined (paratypes).

China. Yunnan: Malipo County, Xiajinchang Township, Laoshan Natural Reserve, 23°07'N, 104°50'E, 1684 m a.s.l., 2 Dec 2015 (no fl. and no fr.), *Q. Fan 13941* (SYS); the same locality, 27 June 2022 (young fr.), *Q. Fan 19580* (SYS); Malipo County, Tianbao Township, Laoshan Natural Reserve, in thick forests on the slopes of limestone hills, 23°11'N, 104°48'E, 1502 m a.s.l., 16 September 2015 (no fl. and no fr.), *Q. Fan 13713* (SYS).

## Supplementary Material

XML Treatment for Eriobotrya crassifolia

## References

[B1] Capella-Gutiérrez S Silla-Martínez JM Gabaldón T (2009) trimAl: A tool for automated alignment trimming in large-scale phylogenetic analyses. Bioinformatics 25(15): 1972–1973. 10.1093/bioinformatics/btp348 19505945 PMC2712344

[B2] Chen SF Meng KK Guo XB Zhao WY Liao WB Fan Q (2020) A new species of *Eriobotrya* (Rosaceae) from Yunnan Province, China. PhytoKeys 146: 61–69. 10.3897/phytokeys.146.50728 32440252 PMC7228930

[B3] Chen SF Milne R Zhou RC Meng KK Yin QY Guo W Ma YP Mao KS Xu KW Kim YD Do TV Liao WB Fan Q (2022) When tropical and subtropical congeners met: Multiple ancient hybridization events within *Eriobotrya* in the Yunnan-Guizhou Plateau, a tropical-subtropical transition area in China. Molecular Ecology 31(5): 1543–1561. 10.1111/mec.16325 34910340

[B4] Dong ZH Qu SH Landrein S Yu WB Xin J Zhao WZ Song Y Tan YH Xin PY (2022) Increasing taxa sampling provides new insights on the phylogenetic relationship between *Eriobotrya* and *Rhaphiolepis*. Frontiers in Genetics 13: e831206. 10.3389/fgene.2022.831206 PMC8964991 35368713

[B5] Gu CZ Spongberg AS (2003) *Eriobotrya*. In: Wu ZY Raven PH Hong DY (Eds) Flora of China Vol.9. Science Press, Beijing, and Missouri Botanical Garden Press, St. Louis, 138–141.

[B6] Idrees M Do TV Gao XF (2018) A new species of *Eriobotrya* (Rosaceae) from Con Dao National Park, southern Vietnam. Phytotaxa 365(3): 288–294. 10.11646/phytotaxa.365.3.6

[B7] IUCN Standards and Petitions Subcommittee (2022) Guidelines for Using the IUCN Red List Categories and Criteria. Version 15. Prepared by the Standards and Petitions Subcommittee. https://www.iucnredlist.org/resources/redlistguidelines

[B8] Kang DH Ong HG Lee JH Jung EK Kyaw NO Fan Q Kim YD (2021) A new broad-leaved species of loquat from eastern Myanmar and its phylogenetic affinity in the genus *Eriobotrya* (Rosaceae). Phytotaxa 482(3): 279–290. 10.11646/phytotaxa.482.3.6

[B9] Katoh K Standley DM (2013) MAFFT Multiple Sequence Alignment Software Version 7: Improvements in Performance and Usability. Molecular Biology and Evolution 30(4): 772–780. 10.1093/molbev/mst010 23329690 PMC3603318

[B10] Li FF Li QY Cui DF Liao WB (2012) *Eriobotryafulvicoma* (Rosaceae), a new species from Guangdong Province, China. Annales Botanici Fennici 49(4): 263–266. 10.5735/085.049.0408

[B11] Liu BB Liu GN Hong DY Wen J (2020a) *Eriobotrya* Belongs to *Rhaphiolepis* (Maleae, Rosaceae): Evidence from Chloroplast Genome and Nuclear Ribosomal DNA Data. Frontiers in Plant Science 10: 1731. 10.3389/fpls.2019.01731 PMC7019104 32117331

[B12] Liu BB Campbell CS Hong DY Wen J (2020b) Phylogenetic relationships and chloroplast capture in the *Amelanchier*-*Malacomeles*-*Peraphyllum* clade (Maleae, Rosaceae): Evidence from chloroplast genome and nuclear ribosomal DNA data using genome skimming. Molecular Phylogenetics and Evolution 147: e106784. 10.1016/j.ympev.2020.106784 32135308

[B13] Nguyen LT Schmidt HA von Haeseler A Minh BQ (2015) IQ-TREE: A fast and effective stochastic algorithm for estimating maximum-likelihood phylogenies. Molecular Biology and Evolution 32(1): 268–274. 10.1093/molbev/msu300 25371430 PMC4271533

[B14] Robertson KR Phipps JB Rohrer JR Smith PG (1991) A synopsis of genera in Maloideae (Rosaceae). Systematic Botany 16(2): 376. 10.2307/2419287

[B15] Yü TT Lu LD Ku TC (1974) Rosaceae. In: Yü TT (Ed.) Flora Reipublicae Popularis Sinicae.Vol. 36. Science Press, Beijing, 260–275.

